# Magnetic Control of Optical Reflectance from Metallic Thin Film Using Surface Plasmon Resonance and Faraday Rotation

**DOI:** 10.3390/ma14123354

**Published:** 2021-06-17

**Authors:** Changjin Son, Heongkyu Ju

**Affiliations:** Department of Physics, Gachon University, Seongnam-si 13120, Gyeonggi-do, Korea; watchmaker@nate.com

**Keywords:** noble metal film, surface plasmon resonance, Faraday rotation, magnetic modulation, magnetic anisotropy, optical reflectance

## Abstract

We demonstrate magnetic control of optical reflectance with no ferromagnetic material via combining the Faraday rotation and the surface plasmon resonance (SPR) in a Kretschman configuration under magnetic fields < 0.5 T. The SPR produces the polarization sensitive reflectance from the Au or Ag thin film coated on a N-BK7 prism in which the Faraday rotation occurs. The gold (Au) or silver (Ag) metal film as a plasmonic film somewhat acts as an incident angle-dependent reflection polarizer that can sensitively sense the polarization change induced by the Faraday rotation that occurs in a prism. We find that combination of Faraday rotation and the surface plasmon can induce a significant magnetic modulation of reflectance normalized with respect to that obtained with no magnetic fields at a specific incident angle of light. The magnetic control of optical reflectance presented may find an application in polarizer-free photonic devices with no ferromagnetic material for magneto-optical modulation.

## 1. Introduction

Magnetic control of photons exploits the interaction between light and magnetism. This interaction that can be activated in magneto-optical materials produces changes in the optical properties including complex refractive indices, resulting in polarizations changes and light attenuation as observed such as in Faraday rotations [1,2,3,4,5,6] and surface magneto-optical Kerr effects [7,8,9].

In particular, the Faraday rotation originates from dielectric responses that are distinct for different handedness of circular polarizations of light propagating in transparent dielectrics under external magnetic fields. Material electron motions driven by electric fields of circular polarizations of light are modified by magnetic Lorentz force in opposite ways between left and right handedness, resulting in a circular birefringence. This enables a linear polarization that can be decomposed into left and right circular ones to rotate non-reciprocally for a give direction of external magnetic fields, forming the basis of optical isolators [10,11,12,13,14].

Surface plasmon resonance (SPR), i.e., the collective oscillation of surface conduction electrons at metal-dielectric (low refractive index dielectric) interface occurs through a phase matching condition between incident photons and surface plasmons into surface plasmon polaritons (SPP) [15,16,17,18]. The condition can be met via evanescent coupling of incident photons through a transparent dielectric of a high index with surface plasmons at the interface. The SPR excitation is extremely sensitive to polarization of incident light since only transverse magnetic (TM) polarizations can support the surface confined modes of electromagnetic fields for surface plasmon polaritons (SPP). In a typical Kretschman configuration where an incident angle is scanned for reflectance versus angle measurements, the reflection minimum accounts for the SPP generation whereby reflected light power is subject to an extreme sensitivity to the polarization of incident light [19,20,21].

Enhancement of Faraday effects have been reported using localized surface plasmons [4,5,6], such as with the noble metal nanoparticles to enhance the magnetic anisotropy of ferromagentic materials. The 8.9 times enhancement of Faraday effects was demonstrated using gold nanowire arrays on the bismuth iron garnet film [14]. However, thin metal film supported SPP has never been combined with Faraday effects for magnetic control of optical properties.

In this work, we report magnetic control of optical reflectance from non-magnetic metal thin film coated on a N-BK7 glass prism under the magnetic fields of medium levels (<0.5 T) at the wavelength of 532 nm. The gold (Au) or silver (Ag) metal film as a plasmonic film somewhat acts as an incident angle-dependent reflection polarizer that can sensitively sense the polarization change induced by the Faraday rotation that occurs in a prism. The magnetic modulations of optical reflectance demonstrated with no ferromagnetic material are unlike those using magneto-optical Kerr effects enhanced by surface plasmons [22,23,24], while similarly exploiting surface plasmon properties and using relatively low magnetic fields (<0.5 T) for modulations of the same order of magnitude of such modulation. This may find an application in non-reciprocal optical devices with no ferromagnetic material nor a polarizer, based on noble metal thin film reflectors with transparent dielectrics.

## 2. Experimentals

**Figure 1:**
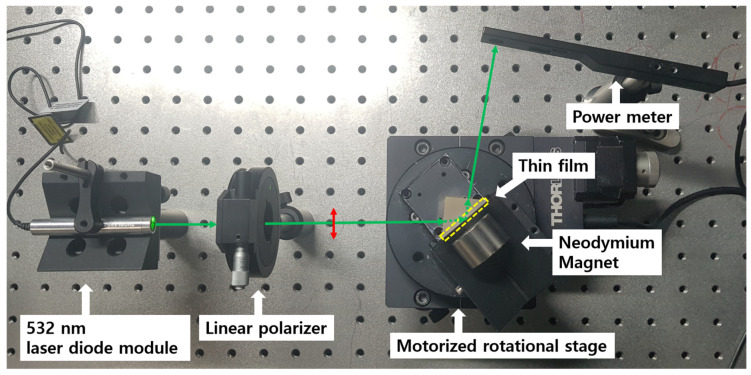
An experimental set up for magnetic control of optical reflectance from the plasmonic metal film coated on a N-BK7 prism using the Faraday rotation.

An experimental setup is shown in Figure 1. Light from a laser diode (CPS 532, Thorlabs Inc, Newton, NJ, USA) at 532 nm wavelength is passed through a linear polarizer (10LP-VIS-B, Newport Inc, Irvine, California, USA) to make nearly horizontal polarization, i.e., the TM polarization as orthogonal to the transverse electric (TE) polarization. Light is then made incident to surface of the metal thin film coated base of a N-BK7 prism at the incidence angle ($\theta_{\mathrm{in}}$) adjusted for SPR, using a motorized rotation stage with the angular resolution of $10^{-4}$ degrees (NR360S, Thorlabs Inc, Newton, NJ, USA). The base surface coated with the noble metal thin film offers a metal-dielectric (air) SPR interface. The plasmonic metal layer, i.e., the 38 nm-thick Au or 50 nm-thick Ag layers, is coated using a thermal evaporator (Daedong Hightech, Gyeonggi-do, Korea) under a working pressure of $6\times 10^{-6}$ Torr.

We use a motorized rotation stage to scan $\theta_{\mathrm{in}}$ with respect to the prism base for measuring reflectance versus $\theta_{\mathrm{in}}$, and obtain the SPR angles corresponding to minimum reflectance, i.e., 48.60° and 44.16° for the Au and Ag thin films. The thicknesses of the noble metal films are determined via optimizing the depth-to-width ratio of the reflectance dip (reflectance minimum) in theoretically simulated reflectance versus $\theta_{\mathrm{in}}$, given the fact that it is proportional to the plasmon resonance quality factor.

A neodymium magnet is added to produce the external magnetic fields normal to the prism base surface as shown in Figure 1. We modulate the magnetic field magnitude from 0 to ~320 mT, that is calibrated by the metal film surface-magnet distance while flipping the field direction by switching the magnet pole. Under various magnetic fields applied, we measure optical power of light reflected from the film coated prism as $\theta_{\mathrm{in}}$ is scanned for respective cases of using the prisms coated with 38 nm-thick Au film and 50 nm-thick Ag film. All experiments are conducted at room temperature.

## 3. Results and Discussion

Propagation of light in the prism prior to reflection from the metal layer produces the linear polarization rotation due to the Faraday rotation for a given magnitude of magnetic fields. Surface normal direction of the magnetic fields permits the polarization rotation to occur in proportion to the surface normal distance (L) between the light entrance point on the prism and the metal surface, i.e., the surface normal projection of the light propagation in the prism. We estimate the prism-generated Faraday rotation angle, i.e., $\theta_{F}$ using a bare N-BK7 prism (with no metal film coating) whose reflected light power is fed to an additional polarizer, i.e., an analyzer with its axis angle adjusted to be along the transverse electric (TE) polarization.

**Figure 2:**
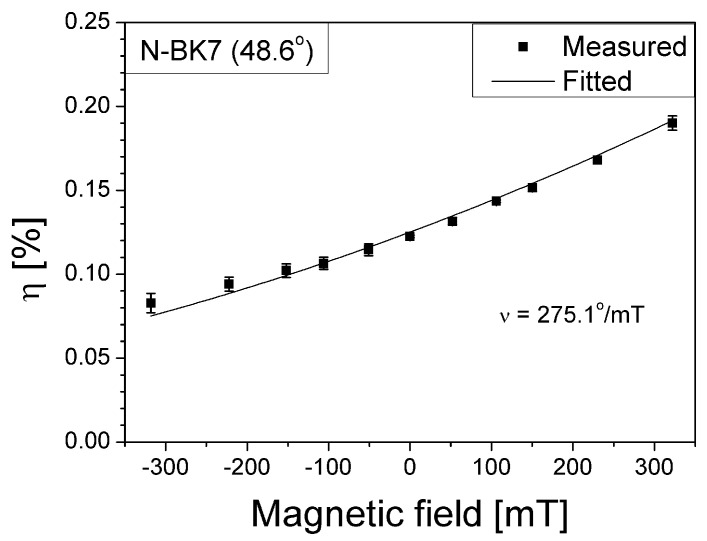
Normalized optical power of light through the analyzer after reflection from a bare prism (no metal coated) under various magnetic field magnitude. The analyzer is TE-polarization oriented while the analyzer output power is normalized with that of light incident to the prism, i.e., η. The positive magnetic fields denote those from the N-poled magnet while negative ones from the S-poled magnet. The plot fitting estimates the Verdet constant ν to be 275.1 °/m·T.

We measure the analyzer output power and normalize it with respect to power of light incident to the prism, i.e., η for various magnetic fields applied (ranging from N 322 mT to S 318 mT) as shown in Figure 2. Fitting to measured data of η versus the magnetic field magnitude enables us to estimate $\theta_{F}$ that varies from −0.725° to 0.734° for an incident angle of light 48.60°, and from −0.785° to 0.795° for an incident angle of 44.16° (reminding that the two different incident angles are those used for the Au and Ag film SPR conditions, respectively). This consequently leads the Verdet constant of the N-BK7 glass prism to be estimated as 275.1 °/m·T, being ~23% lower than that of BK7 glass as found in [25,26].

When replacing the bare prism with the metal thin film coated one for combining the SPP effects with the Faraday rotation, we remove the analyzer from the set-up and make the input polarizer axis 2.1° tilted from the horizontal direction, thus incident light having slight components of TE polarizations. We expect that the small tilted angle of the polarizer from the TM direction enables SPP to produce significant attenuation of reflected light, though the Faraday rotation of about 0.8° (calculated) occurs. The angle that needs to be tilted by more than 0.8° thus allows the polarization-sensitive SPP to produce detectable difference between increase and decrease in reflectance due to the opposite directions of Faraday rotation caused by opposite directions (N and S poled magnets) of magnetic fields. The zero tilted angle would otherwise always increase reflectance irrespective of directions of magnetic fields due to the features of SPP that attenuates reflectance of TM polarized light only. It is therefore expected that, with the nonzero small tilted angle, the reflectance change induced by Faraday rotation, though slight in magnitude, can be visible when normalized with respect to reflectance obtained (as a small as less than 1% in magnitude) with no magnetic fields. Further Faraday rotation occurs after reflection, adding up to the pre-reflection polarization rotation due to the magneto-optical non-reciprocity.

**Figure 3:**
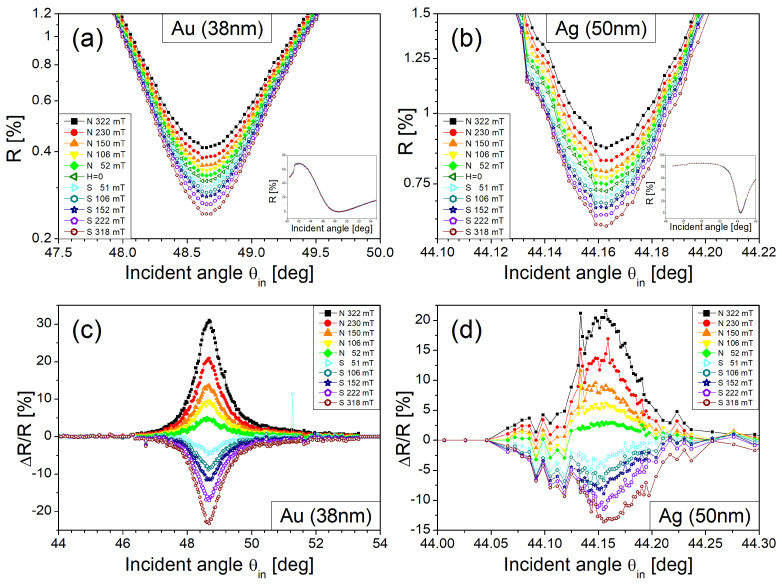
Optical reflectance (R) and the normalized reflectance change (ΔR/R) versus incident angle θ_in_. The R obtained with 38 nm thick Au film and with 50 nm thick Ag film is given in (**a**,**b**), respectively, while ΔR/R for 38 nm-thick Au film and 50 nm-thick Ag film given in (**c**,**d**), respectively.

Figure 3a presents the optical reflectance (R) for the 38 nm-thick Au film. The reflectance is shown at around the SPR angle under various magnetic fields, while its inset providing the reflectance over all $\theta_{\mathrm{in}}$ scanned with no magnetic fields. It is observed that the reflectance changes with no SPR angle alteration as the magnetic field’s magnitude varies. The fact that the magnetic fields applied change no SPR angle implies that the SPP wave vectors are not altered. This indicates that the Au thin film is not magnetic enough to generate surface magneto-optical Kerr effects (polar mode) under the magnetic fields applied (<0.5 T), though the magnetic anisotropy possibly expected to be activated by its surface plasmonic enhancement based on a pure Au thin film, which is unlike its plasmonic enhancement using the nanosized Au disks [27,28,29]. The feature of the negligible magnetism found in the metal film itself is also found in the Ag film as seen in Figure 3b.

The magnetic modulation of reflectance (ΔR) becomes larger at an angle closer to the SPR angle. It becomes clearer in Figure 3c,d where the normalized reflectance (ΔR/R) is given as a function of $\theta_{\mathrm{in}}$. In a case of the 50 nm-thick Ag film, the similar features are found with noisier characteristics as seen in Figure 3d. The noisier properties are partly due to intrinsic reflectance modulation under no magnetic fields which occurs with its magnitude relatively smaller (over an angle range of 0.1°) in the Ag film case than in the Au film (over an angle range of 2°). Another possible cause for such noise is the surface roughness of the Ag film (its rms of 2.1 nm) higher than that of the Au film (its rms of 1.1 nm).

ΔR/R reaches maximum of about 30% and 20% at 322 mT (N-poled magnet) for the Au and Ag thin films, respectively, as shown in Figure 3b,d. It is also observed that the slight asymmetry in ΔR/R occurs between magnetic fields from N and S-poled magnets. This asymmetry mainly stems from the following: the Faraday rotation linearly proportional to the magnetic field magnitude modulates nonlinearly the TM polarization component of an electric field of light incident to the metal film, since it is proportional to cosine (2.1° $+\theta_{F}$).

The magnetic modulation of optical reflectance demonstrated above can be simulated using the Faraday rotation combined with SPR induced polarization sensitivity of reflectance from the plasmonic film. The Jones matrix expression used for simulation is

(1) $$\left(\begin{array}{c} E_{TM}^{\prime }\\ E_{TE}^{\prime } \end{array}\right)=R\left(\theta_{F}\right)\left(\begin{array}{cc} r_{spr}\left(\theta_{in}\right) & 0\\ 0 & e^{i\delta } \end{array}\right)R\left(\theta_{F}\right)\left(\begin{array}{c} E_{TM}\\ E_{TE} \end{array}\right)$$

Here $E_{TM}$ and $E_{TE}$ are the normalized TM and TE components of electric fields of light incident to the prism, and $R\left(\theta_{F}\right)$ is the Faraday rotation matrix where $\theta_{F}=\nu LB$ (B is the magnetic induction). Here $r_{spr}$ is the TM reflection coefficient with its magnitude obtained from the measured reflectance (Figure 3a,b). Here the phases of reflection coefficients ($r_{spr}$ and $e^{i\delta }$) for TE and TM polarizations are theoretically obtained from the Fresnel reflection formula of the three layers (the prism-the metal film-air). It is noted that an input polarizer imperfection in polarization filtering would produce an elliptical polarization highly eccentric towards the linear polarization tilted by 2.1° from a horizontal direction. Numerical fit to measured transmission of light through both the 2.1° tilted input polarizer and an angle-scanned analyzer permits us to obtain $E_{TM}=\cos\Delta \theta$, $E_{TE}=\sin\Delta \theta e^{i\alpha }$ where Δθ ~ 2.1° and α ~ 0.97.

**Figure 4:**
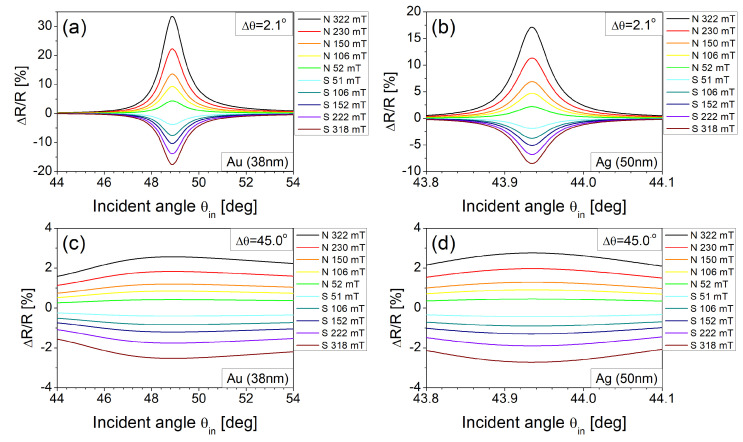
Numerical simulation of normalized reflectance ΔR/R versus incident angle $\theta_{\mathrm{in}}$ under various magnetic fields for the different tilted angles (Δθ) of an input polarizer. (**a**) the Au film at Δθ = 2.1°, (**b**) the Ag film at Δθ = 2.1°, (**c**) the Au film at Δθ = 45.0°, and (**d**) the Ag film at Δθ = 45.0°.

With the parameters ν, Δθ and α obtained above, ΔR/R is simulated as shown in Figure 4a,b at incident angles around the SPR angle for the Au and Ag films, respectively. The magnetic modulations of reflectance simulated show qualitatively good agreement with the measured ones. Moreover, it is visible that tilting the input polarizer by 45° to have equal optical power between TE and TM components smears out magnetic modulation as seen in Figure 4c,d, as expected earlier.

## 4. Conclusions

We present the magnetic control of optical reflectance using a polarization sensitive SPR and the Faraday rotation in a Kretschman configuration with magnetic fields. Under magnetic fields < 0.5 T, the Faraday rotation occurs during propagation of light inside the prism while the plasmonic thin films of noble metals coated on the prism base produce the polarization-sensitive reflectance via SPR. An appropriate tuning of polarization of light incident to the prism can greatly enhance the magnetic modulation of reflectance normalized with that obtained with no magnetic field. In addition, it is observed that the magnetic fields applied make no change in SPR angles for the Au or Ag thin film. This indicates that the plasmonic enhancements of surface magneto-optical Kerr effects in such noble metal thin films are negligible under such medium levels of magnetic fields. However, we find that the combination of SPP and Faraday rotation can induce magnetic modulation of optical reflectance without a ferromagnetic material, being maximized at a specific incident angle of light. The magnetic control of optical reflectance presented may find an application in polarizer-free photonic devices with no ferromagnetic material for magneto-optical modulation.

## Data Availability

The data presented in this study are available on request from the corresponding author. The data are not publicly available due to privacy.
